# Application of the Actiphage® Assay to Detect Viable *Mycobacterium avium* subsp. *paratuberculosis* Cells in Fresh Sheep and Goat Milk and Previously Frozen Milk and In-Line Milk Filters

**DOI:** 10.3389/fvets.2021.752834

**Published:** 2021-10-11

**Authors:** Monika Beinhauerova, Iva Slana

**Affiliations:** ^1^Department of Microbiology and Antimicrobial Resistance, Veterinary Research Institute, Brno, Czechia; ^2^Department of Experimental Biology, Faculty of Science, Masaryk University, Brno, Czechia

**Keywords:** *Mycobacterium avium* subsp. *paratuberculosis*, paratuberculosis, phage-based detection, milk, frozen samples, Actiphage, viability determination, milk filter

## Abstract

*Mycobacterium avium* subsp. *paratuberculosis* (MAP) is a well-known causative agent of paratuberculosis, a chronic infectious granulomatous enteritis of ruminants contributing to significant economic losses worldwide. Current conventional diagnostic tools are far from being sufficient to manage and control this disease. Therefore, increased attention has been paid to alternative approaches including phage-based assays employing lytic bacteriophage D29 to detect MAP cells. The aim of the present study was to assess the applicability and efficiency of the recently developed phage-based kit termed Actiphage® combined with IS*900* real-time PCR (qPCR) for rapid detection and quantification of viable MAP in milk samples. We demonstrated that Actiphage® in combination with IS*900* qPCR allows for rapid and sensitive detection and identification of viable MAP in milk samples with a limit of detection of 1 MAP per 50 ml milk. Using this method, the presence of viable MAP cells was successfully determined in 30.77% of fresh goat, sheep and cow milk samples originating from paratuberculosis-affected herds. We further used Actiphage assay to define the time-lapse aspect of testing naturally contaminated milk and milk filters frozen for various lengths of time by phage-based techniques. Viable MAP was detected in 13.04% of frozen milk samples and 28.57% of frozen milk filters using Actiphage-qPCR. The results suggest the ability to detect viable MAP in these samples following freezing for more than 1 year. The obtained results support the views of the beneficial role of this technology in the control or monitoring of paratuberculosis.

## Introduction

*Mycobacterium avium* subsp. *paratuberculosis* (MAP) is the etiological agent of paratuberculosis (or Johne's disease) manifesting after a long latent period particularly in domestic and wild ruminants, e.g., cattle, sheep, goats and deer. Clinical symptoms include chronic weight loss and wasting, diarrhoea, reduced milk yield, and animal death from exhaustion (1). However, animals with clinical symptoms represent only a small proportion of the infected animals within a herd. Carriers of MAP with a subclinical form of the disease can persist in a herd undetected for years and transmit MAP to healthy individuals *via* the faecal-oral route or through milk with secreted MAP to new-born animals (2). Paratuberculosis is widespread throughout the world and herd-level prevalence rates are increasing globally in food-producing animals, causing significant economic losses annually (3, 4). Deficiencies in commonly used diagnostic tests (e.g., culture, PCR, ELISA), such as inaccuracy in the early stage of infection, the long period of time required for result acquisition and the inability of live-dead discrimination, are the major reasons why effective control of paratuberculosis is problematic. Therefore, efforts have been focused on the development of alternative diagnostic approaches such as the phage amplification assay (PA), incorporation of which in diagnostic strategies of paratuberculosis could contribute to more reliable animal infection status assessment and, ultimately, to a reduction in the prevalence of MAP-infected individuals in herds (5).

PA technology exploits the natural life cycle of lytic bacteriophages, during which the bacteriophages replicate only within their target host cells, and productive infection is detectable through plaque formation in a lawn of non-pathogenic *M. smegmatis* on an agar plate. In view of the fact that the bacteriophage D29 used in PA technology does not infect MAP alone, but is known for its broader range of hosts within the genus *Mycobacterium* (6), the identity of detected mycobacterial cells is confirmed by endpoint analysis using plaque PCR targeting the IS*900* signature sequence specific for MAP (7). Since its first description, PA has been successfully used for viable MAP detection and enumeration in a range of matrices including raw milk (1, 7–10), pasteurised milk (11), cheese (12), calf milk replacer (13), powdered infant formula (14), blood (15) and faeces (8). Based on satisfactory study results, PA has been proposed for clinical and food samples examination for the presence of viable MAP in place of conventional culture to speed up result acquisition (within 2 days instead of 6 weeks or more). Moreover, no chemical decontamination of samples is required in the case of PA, giving PA enhanced detection sensitivity compared to culture (7). In order to achieve almost 100% correlation between plaque and colony counts, the PA method was optimised allowing accurate quantification of viable MAP cells with a detection limit of approximately 10 MAP CFU/ml of milk (4). In addition to MAP detection, PA has also proven to be effective in the diagnosis of bovine tuberculosis, where it allowed to reveal mycobacteremia of animals from a single intradermal comparative cervical tuberculin (SICCT)-positive cattle herd in combination with recombinase polymerase amplification (16).

The plate-based format of PA has recently been modified and a commercial kit termed Actiphage® Rapid (PBD Biotech, Suffolk, UK), maintaining the sample in a single tube and enabling high-throughput sample testing, has been developed (17). Actiphage utilises bacteriophage lytic ability allowing mycobacterial DNA to be effectively released and detected by PCR. This approach was shown to be more sensitive (limit of detection ≤ 10 cells/ml of blood) and less laborious than the original PA in detecting MAP since it eliminates the need for agar preparation and plaque examination, and the results are available within 6 h (17).

The objective of the present study was to evaluate the recently developed Actiphage assay in combination with IS*900* real-time PCR (qPCR) for rapid detection of viable MAP cells in milk samples of various origin. For this purpose, the limit of detection of Actiphage-IS*900* qPCR in milk was established in the first instance. Following this determination, goat, sheep and cow milk samples originating from naturally MAP-infected herds were investigated by Actiphage-qPCR, with the results being compared with those obtained by direct IS*900* qPCR. Furthermore, the effect of sample storage and freezing on Actiphage-qPCR performance was analysed on milk-based samples previously tested positive by direct IS*900* qPCR and the results were compared to conventional PA.

## Materials and Methods

### Preparation of MAP Suspension for Spiking Experiments

A MAP strain (field isolate 7072 originating from a white deer) was used throughout the study. The isolate was cultured on Herrold's egg yolk medium (HEYM) supplemented with 2 mg/ml Mycobactin J (Allied Monitor, USA) at 37°C for 4 weeks. The colonies were harvested using a loop and resuspended in 1.2 ml of Middlebrook 7H9 broth (M7H9; Difco Laboratories, USA) with 10% Middlebrook OADC enrichment (Difco Laboratories). 350 mg of 1 mm zirconia/silica beads (Biospec, USA) was added to the suspension, followed by vortex homogenisation for 30 s. The suspension was centrifuged at 100 g for 30 s to remove large MAP clumps and the upper cell fraction was resuspended in M7H9 broth and diluted to an optical density at 600 nm (OD_600_) of 0.15–0.20 corresponding to ~10^8^ MAP cells/ml of suspension (18). This suspension was further 10-fold serially diluted in a range from 10^6^ to 10^2^ CFU/ml and used for the spiking of prepared milk pellets.

The amount of MAP cells in each dilution was quantified by IS*900* qPCR. In brief, 400 μl of each dilution was transferred into 2-ml screw cap tubes containing 350 mg of 0.1 mm zirconia/silica beads (Biospec) and the cells were disrupted in a MagNA Lyser (Roche Molecular Diagnostic, Germany) at 7,000 rpm for 60 s. After centrifugation at 14,000 g for 60 s, the supernatant was collected and used as a template for in-house IS*900* qPCR (19). Quantification by qPCR was performed in parallel to the artificially contaminated milk pellets after undergoing the Actiphage assay.

### Sample Collection and Processing

The MAP negative milk samples (bulk tank sheep and goat) used in spiking experiments for protocol validation originated from a herd in the Czech Republic that has never had any known or suspected presence of paratuberculosis.

MAP naturally positive fresh milk samples (individual or bulk tank milk; *n* = 13) originated from Czech sheep, goat and cow farms with a known history of paratuberculosis. Previously acquired naturally contaminated milk samples (individual or bulk tank milk; *n* = 23) from sheep, goat and cow farms in the Czech Republic and Germany were stored at −70°C for various lengths of time (see **Table 3**). Textile milk filters (*n* = 14), which are part of milking devices preventing coarse foreign matter from migrating to the milk tanks, were obtained from dairy cow farms within the Czech Republic and were stored at −70°C in sterile plastic bags for 1 or 7 years. All these frozen samples (milk and milk filters) were tested positive within the paratuberculosis control program by direct IS*900* qPCR using the protocol described by Slana et al. (19), with IS*900* qPCR being performed immediately after the samples were freshly collected.

50-ml milk samples (fresh or frozen) were centrifuged in a conical centrifuge tube at 2,500 g at room temperature for 15 min. The cream layer and supernatant were removed leaving a pellet undisturbed in the bottom of the tube. The pellet was resuspended in 3 ml of Actiphage Medium Plus (PBD Biotech Ltd, UK) followed by centrifugation at 2,500 g at room temperature for 10 min. The supernatant was removed. The milk samples processed in this way were then subjected to the Actiphage-qPCR assay or conventional PA. In the case of frozen milk samples, these assays were preceded by the milk pellets being resuspended in 1 ml of Actiphage Medium Plus and incubated overnight at 37°C, followed by centrifugation at 13,000 g for 3 min and supernatant removal.

Textile milk filters were cut into strips around 5 mm wide and 1.5 cm long using sterile scissors. Ten such strips (equal to 1 g) and 10 ml of PBS were added to 50-ml centrifuge tubes with 15 sterile 3.5 mm glass beads (Biospec). The samples were then mechanically homogenised on a vortex for 30 s at maximum speed, and the suspensions were transferred to a new centrifugation tube and centrifuged at 2,500 g for 10 min. The supernatant was discarded and the pellet was resuspended in 1 ml of Actiphage Medium Plus. Following overnight incubation at 37°C, the samples were homogenised by vortexing and divided into two equal parts, one of which was tested by the Actiphage-qPCR assay and the other by conventional PA. Before performing the Actiphage-qPCR assay or conventional PA, the samples were centrifuged at 13,000 g for 3 min to recover MAP and the supernatant was removed.

### Determining the Limit of Detection of Actiphage-IS*900* qPCR in Milk

Mixed sheep and goat milk samples that were proven negative for the presence of MAP cells by IS*900* qPCR were artificially contaminated with five prepared dilutions containing 5 × 10^5^-1 × 10^2^ MAP cells/ml quantified by IS*900* qPCR. To each of the two tubes with prepared milk pellet, 10 μl of the respective MAP suspension diluted 10-fold was added and the Actiphage-qPCR assay was performed. The experiment was carried out in two independent repetitions. The LOD for the Actiphage-qPCR was determined as the lowest theoretical amount of MAP cells that was possible to detect in all replicates regardless of the absolute quantity.

### Actiphage Assay and Purification of Mycobacterial DNA

MAP detection using the Actiphage® Rapid Kit (PBD Biotech Ltd; distributed by BioSellal, Dardilly, France) was performed according to the manufacturer's instructions. In brief, pre-processed milk pellets and milk filters were resuspended in 200 μl of Actiphage Medium Plus and transferred to a clean 2-ml screw cap tube. Then 20 μl of Actiphage Reagent, which was prepared by the reconstitution of the lyophilised Actiphage vial in 220 μl of Actiphage Medium Plus, was added to each sample followed by incubation at 37°C for 3.5 h. After centrifugation at 13,000 g for 3 min, the supernatants were transferred into the top filter section of individual Rapid Tubes and again centrifuged at 13,000 g for 3 min. The filter section of the Rapid Tube was removed and the flow through in the bottom of the tube containing mycobacterial genomic DNA was purified before qPCR. The purification of mycobacterial genomic DNA was carried out using the PurePhage® Kit Protocol according to the manufacturer's instructions (BioSellal) with the exception of elution, which was performed up to a volume of 25 μl, and 5 μl of purified DNA was used as a template for IS*900* qPCR.

Positive and negative controls were included during each test performed according to the manufacturer's instructions.

### Conventional Phage Amplification Assay

The conventional PA was performed as previously published (4) with minor modifications. Processed milk pellets and milk filters were resuspended in 1 ml of M7H9 broth supplemented with 10% Middlebrook OADC enrichment, 2 mM CaCl_2_ (Penta, Czech Republic) and 1.25% PANTA (Becton Dickinson, USA). Subsequently, 100 μl of bacteriophage D29 (kindly provided by Dr Botsaris, Cyprus University of Technology, Cyprus and Dr Rees, University of Nottingham, UK; stock concentration 10^9^ PFU/ml) was added to the sample and incubated at 37°C for 2 h with shaking at 100 rpm. The bacteriophages that had not infected mycobacterial cells were subsequently destroyed by the addition of 100 μl of 100 mM ferrous ammonium sulphate solution (FAS; Lach-Ner, Czech Republic). After incubation at room temperature for 5 min, the FAS was neutralised by the addition of 5 ml of enriched medium followed by preparation of 10-fold serial dilutions of samples. One ml of *Mycobacterium smegmatis* mc^2^155, grown for 48 h at 37°C with shaking at 100 rpm in 50 ml of M7H9 broth with 10% OADC (10^8^ CFU/ml), was added to each sample dilution. Each of the samples was transferred to a sterile Petri dish and 6 ml of molten 1.6% Middlebrook 7H10 agar (Difco Laboratories) cooled to 55°C was added. The content of the plate was thoroughly mixed and left at room temperature until the agar set. The number of plaques formed was counted after overnight incubation of the plates at 37°C. As experimental controls, a 1 ml sample containing ~1 × 10^2^ CFU/ml *M. smegmatis* cells and 1 ml of enriched medium alone were used as positive and negative controls, respectively.

The identity of mycobacteria detected was analysed by plaque PCR (7), and up to 20 plaques per sample were examined. The centre of the plaque was excised using a sterile loop and transferred to 10 μl of ultrapure water (Top-Bio, Czech Republic), heated at 95°C until the agar melted, and immediately frozen at −20°C. After 15 min at this temperature, the samples were thawed and DNA present in the supernatant was analysed by in-house IS*900* qPCR (19).

### Isolation of MAP DNA From Milk

The milk samples were examined for the presence of MAP DNA using a slightly modified protocol from the Quick-DNA Faecal/Soil Microbe Microprep Kit (Zymo Research, USA). Initially, 50 ml of milk samples were centrifuged in a conical centrifuge tube at 4,200 g at 15°C for 45 min. The cream layer and supernatant were removed leaving the pellet undisturbed in the bottom of the tube. The pellet was resuspended in the residual supernatant remaining in the tube (~1 ml) and the suspension was transferred to a 2-ml screw cap tube followed by centrifugation at 14,500 g for 10 min. The supernatant was discarded and the pellet was dissolved in 750 μl of BashingBead Buffer and transferred into a BashingBead Lysis Tube. Subsequently, the sample was subjected to mechanical homogenisation in a MagNA Lyser at 6,400 rpm for 1 min. Following centrifugation at 10,000 g for 1 min, 400 μl of the supernatant was processed using an isolation kit according to the manufacturer's instructions. The DNA was eluted into 25 μl of DNA Elution Buffer and used as a template for the in-house IS*900* qPCR assay.

### Amplification of the IS*900* Signature Sequence

Detection and quantification of MAP cells was performed on a LightCycler 480 instrument (Roche Molecular Diagnostic, Germany) using an in-house IS*900* qPCR assay (19) or a commercial Bio-T kit® (BioSellal). Each sample was run in a technical duplicate for both qPCR assays using the same thermocycler settings (19). In the case of the in-house IS*900* qPCR assay, the MAP cell quantity was determined using the “fit-point analysis” option of the LightCycler 480 software (version 1.5.1.62) according to a calibration curve derived from a plasmid standard gradient containing the IS*900* qPCR product insert. In the case of qPCR using the Bio-T kit, qualitative assessment of MAP presence in samples was performed.

### Optimisation of Plaque PCR

In view of repeated plaque PCR negative results of real samples, which produced plaques on an agar plate, optimisation experiments were performed comparing three different plaque PCR procedures to ensure elimination of false negative results. In these experiments, plaques obtained from the MAP positive control of a conventional PA were used. The first procedure (Approach A) was based on the freezing-thawing method published by Stanley et al. (7) and is described above in the paragraph “Conventional phage amplification assay.” The second procedure (Approach B) involved the addition of 250 μl of Tris-EDTA buffer (Amresco, USA) and 350 mg of 0.1 mm zirconia/silica beads to a 2-ml screw cap tube with an excised plaque and homogenisation on a MagNA Lyser at 7,000 rpm for 5 s. Samples were subsequently centrifuged at 14,500 g for 3 min and 5 μl of supernatant was analysed by in-house IS*900* qPCR. During the third procedure (Approach C), the commercial Zymoclean Gel DNA Recovery Kit (Zymo Research) was used according to the manufacturer's instructions; DNA was eluted into 10 μl of DNA Elution Buffer and quantified by in-house IS*900* qPCR. In order to exclude the inhibition of plaque PCR, detection of *M. smegmatis* mc^2^155, which should be present in each plaque, was evaluated by plaque PCR targeting the mycobacterial internal transcribed spacer (ITS) locus using the primer set and probe described previously (20).

## Results

### Determining the Limit of Detection of Actiphage-IS*900* qPCR in Milk

The LOD of Actiphage was determined using MAP-negative milk samples artificially contaminated with a serially diluted MAP suspension (Table 1). The Actiphage assay in combination with in-house IS*900* qPCR enabled to detect even the lowest theoretical amount of MAP cells used to spike milk pellets (1.00 × 10^0^) in all four replicates. The LOD was, therefore, set to this value.

**Table 1:** Determining the limit of detection of the Actiphage assay in milk.

**Theoretical amount of MAP cells^a^**	**Real data from qPCR—No.** **of replicate**			
	**1**	**2**	**3**	**4**
5.00 × 10^3^	+	+	+	+
5.00 × 10^2^	+	+	+	+
5.00 × 10^1^	+	+	+	+
5.00 × 10^0^	+	+	+	+
1.00 × 10^0^	+	+	+	+

a *Quantification of Mycobacterium avium subsp. paratuberculosis (MAP) cells added to milk pellets was based on IS900 qPCR analysis*.

### The Fresh Milk Samples Testing by Actiphage-IS*900* qPCR

Following LOD determination in milk, the applicability of Actiphage-IS*900* qPCR was evaluated by testing naturally contaminated milk samples. The analysis using Actiphage-qPCR enabled to detect viable MAP cells in a total of 4 (30.77%) of 13 milk samples (Table 2) with MAP numbers detected ranging from 4.10 × 10^0^ to 1.33 × 10^1^ per 50 ml milk. In contrast, direct IS*900* qPCR detected MAP in 10 (76.92%) of these samples with MAP cell quantity estimates ranging from 1.00 × 10^0^ to 2.11 × 10^1^ per 50 ml milk. All samples tested positive by Actiphage-qPCR also gave a positive result by direct IS*900* qPCR, suggesting that no false positive results were obtained by the Actiphage-qPCR assay.

**Table 2:** Comparison of the Actiphage-qPCR assay with direct IS*900* qPCR on fresh milk samples.

**Sample no**.	**Sample origin**	**Characteristics**	**Direct IS*900* qPCR (copy/50 ml)**	**Actiphage-IS*****900*** **qPCR**	
				**In-house qPCR (copy/50 ml)**	**Bio-T kit**
1	sheep	bulk tank milk	7.97 × 10^0^	1.01 × 10^1^	+
2			1.00 × 10^0^	-	-
3	goat	bulk tank milk	1.10 × 10^1^	1.33 × 10^1^	+
4			1.51 × 10^1^	–	+
5	cow	individual milk	1.09 × 10^1^	–	–
6			–	–	–
7			1.41 × 10^0^	–	–
8			1.78 × 10^0^	–	–
9			–	–	–
10			3.93 × 10^0^	–	–
11			–	–	–
12			2.11 × 10^1^	4.10 × 10^0^	–
13			1.28 × 10^1^	–	–

–* denotes a negative result, + denotes a positive result*.

### The Time-Lapse Aspect of Testing Frozen Milk-Based Samples by the Actiphage Assay

In order to determine the effect of freezing the sample for various lengths of time (up to 13 years) on the effectiveness of the Actiphage-qPCR assay, 37 frozen milk-based samples were subjected to analysis by Actiphage-qPCR assay and the results were compared with those of conventional PA (Table 3). Of 23 frozen milk samples, 3 (13.04%) tested positive by the Actiphage-qPCR assay. Two were sheep and goat bulk tank milk samples frozen for 14 days. Another positive sample was one bulk tank cow milk frozen for seven years. Of 14 frozen milk filters, 4 (28.57%) gave a positive result by the Actiphage-qPCR assay (all milk filters frozen for 1 year). Overall, more positive records were obtained using a Bio-T kit than using in-house IS*900* qPCR as the endpoint assay. The MAP load in these samples determined by Actiphage-qPCR (8.02 × 10^0^-9.18 × 10^0^ cells per 50 ml milk and 2.25 × 10^0^ cells per 1 g of milk filter) roughly corresponded to quantities obtained by direct IS*900* qPCR (1.00 × 10^0^-4.93 × 10^2^ cells per 50 ml milk and 1.09 × 10^0^-4.68 × 10^3^ cells per 1 g of milk filter). Regarding the conventional PA, plaques were produced in 11 (47.83%) of 23 frozen milk samples (range: 2.18 × 10^0^-1.09 × 10^4^ PFU per 50 ml milk) and in 3 (21.43%) of 14 frozen milk filters (range: 2.18 × 10^1^-1.74 × 10^2^ PFU per 1 g of milk filter).

**Table 3:** Evaluation of Actiphage-qPCR performance on naturally contaminated milk-based samples frozen for different time periods.

**Sample no**.	**Sample origin**	**Characteristics**	**Time frozen**	**Direct IS*900* qPCR^a^ (copy/50 ml or 1 g)**	**Conventional PA (PFU/50 ml or 1 g)**	**Actiphage^b^ (copy/50 ml or 1 g)**
1	sheep	bulk tank milk	14 days	7.97 × 10^0^	n. a.	8.02 × 10^0^
2			1 month	1.00 × 10^0^	3.27 × 10^3^	–
3			1 year	7.97 × 10^0^	3.38 × 10^3^	–
4	goat	bulk tank milk	14 days	1.10 × 10^1^	n. a.	9.18 × 10^0^
5			1 month	1.51 × 10^1^	–	–
6			1 year	1.10 × 10^1^	1.09 × 10^4^	–
7	cow	bulk tank milk	7 years	1.00 × 10^1^	4.36 × 10^1^	–
8			7 years	1.15 × 10^1^	1.09 × 10^1^	(+)
9			11 years	1.10 × 10^2^	–	–
10			11 years	5.95 × 10^1^	–	–
11			11 years	1.28 × 10^2^	–	–
12			11 years	9.70 × 10^1^	–	–
13	cow	individual milk	6 years	3.77 × 10^1^	3.49 × 10^3^	–
14			13 years	4.77 × 10^2^	2.18 × 10^0^	–
15			13 years	4.93 × 10^2^	2.18 × 10^0^	–
16			13 years	1.12 × 10^2^	1.50 × 10^2^	–
17			13 years	9.90 × 10^1^	–	–
18			13 years	2.20 × 10^2^	8.68 × 10^2^	–
19			13 years	2.85 × 10^2^	–	–
20			13 years	7.30 × 10^1^	–	–
21			13 years	1.68 × 10^2^	–	–
22			13 years	3.38 × 10^2^	4.36 × 10^1^	–
23			13 years	2.50 × 10^2^	–	—
24	cow	milk filter	1 year	4.23 × 10^0^	–	–
25			1 year	1.58 × 10^0^	–	–
26			1 year	2.54 × 10^0^	–	–
27			1 year	7.36 × 10^0^	–	2.25 × 10^0^
28			1 year	1.09 × 10^0^	–	–
29			1 year	6.54 × 10^0^	–	(+)
30			1 year	1.52 × 10^0^	–	(+)
31			1 year	5.06 × 10^0^	–	–
32			1 year	3.51 × 10^0^	–	–
33			1 year	4.23 × 10^0^	–	–
34			1 year	5.45 × 10^1^	8.72 × 10^1^	–
35			1 year	1.13 × 10^1^	–	(+)
36			7 years	2.29 × 10^2^	2.18 × 10^1^	–
37			7 years	4.68 × 10^3^	1.74 × 10^2^	–

*–denotes a negative result, + denotes a positive result*.

a *Direct IS900 qPCR was performed immediately after the samples were freshly collected*.

b *Results obtained by Actiphage in combination with in-house IS900 qPCR or using a commercial Bio-T kit (in round brackets); n. a., not analysed*.

### Optimisation of the Plaque PCR

All three DNA extraction approaches tested were found to be efficient enough to enable the detection of MAP DNA originating from a single plaque using subsequent IS*900* qPCR (Table 4). However, only the commercial Zymoclean Gel DNA Recovery Kit (Approach C) provided an approximately three-fold and five-fold increase in the IS*900* signal when 3 and 5 plaques, respectively, were used. In addition, *M. smegmatis* was detected by targeting the ITS locus of the DNA in each plaque evaluated excluding inhibition of plaque PCR. We also evaluated the effect of DNA purification using the PurePhage kit (BioSellal) on plaque PCR performance; however, no significant increase in DNA yield was achieved (data not shown).

**Table 4:** Performance of different plaque PCR approaches in detecting the IS*900* signature sequence in DNA extracted from various numbers of plaques.

**No. of plaques**	**Mean IS*****900*****/5** **μl**		
	**Approach A^a^**	**Approach B^b^**	**Approach C^c^**
1	5.77 × 10^1^	3.44 × 10^1^	7.31 × 10^1^
3	6.86 × 10^1^	5.86 × 10^1^	2.46 × 10^2^
5	9.56 × 10^1^	1.49 × 10^2^	3.66 × 10^2^

a *The procedure based on the freezing-thawing method published by Stanley et al. (7)*.

b *The in-house procedure based on homogenisation of plaques in a MagNA Lyser instrument with the addition of Tris-EDTA buffer and 0.1 mm zirconia/silica beads followed by centrifugation*.

c *The commercial Zymoclean Gel DNA Recovery Kit (Zymo Research)*.

## Discussion

This study focused on performance evaluation of the recently developed phage-based assay known as Actiphage combined with IS*900* qPCR for rapid detection and quantification of viable MAP cells in milk samples of various origins. To date, only blood investigations for the presence of viable mycobacteria using the Actiphage assay have been described, with the method being successfully applied for the detection of MAP bacteremia in experimentally infected cattle (17) and newborn calves (21). *M. tuberculosis* complex bacteremia was identified by Actiphage in naturally infected SICCT-positive cattle (17) and in immunocompetent patients with active and incipient tuberculosis (22), also reflecting the great potential of this method in the early diagnostics of pulmonary tuberculosis. Phage-based methods providing viability information are of great importance in the diagnosis of mycobacterial diseases, especially in paratuberculosis, in which the application of conventional cultivation methods for viability determination is hampered by very long incubation times (23). First, the sensitivity of Actiphage-IS*900* qPCR in milk was established using milk pellets artificially contaminated by serial MAP dilutions with cell numbers quantified by qPCR with results indicating the ability to detect even trace amounts of viable MAP cells in milk (LOD ≈ 1 MAP cell). This finding suggests a sensitivity about an order of magnitude higher than that achieved in the case of conventional PA (4, 24) and at least two orders of magnitude higher as compared to a conventional culture (25). The results obtained are in accordance with the study evaluating the applicability of Actiphage for detecting MAP bacteremia in cattle populations in which the determined LOD value was shown to be ≤ 10 cell ml^−1^ of blood (17).

Having established the LOD of Actiphage-IS*900* qPCR in milk, we proceeded to test 13 fresh milk samples originating from herds naturally infected with MAP to assess the applicability of the method on naturally contaminated samples. The presence of viable MAP cells was successfully detected in a total of four fresh milk samples (30.77%), indicating that an optimised protocol involving milk sample preparation and subsequent Actiphage-IS*900* qPCR testing is appropriate for diagnostic purposes. In contrast to Actiphage-IS*900* qPCR, direct IS*900* qPCR detected MAP DNA in 10 milk samples (76.92%). Considering bacteriophage D29 capability to replicate only within viable mycobacterial cells (4), a possible explanation as to why six direct IS*900* qPCR-positive samples were not tested positive by one of the Actiphage-qPCR approaches may be that only dead MAP cells were present in the milk samples. Moreover, given that samples were recorded positive for all three types of milk, it seems that none of milk components interfere with the phage during testing. It has previously been observed that free MAP cells are predominantly present in the cream fraction of milk due to their strongly lipophilic nature (26). Lipids were, however, shown to inhibit phage infection, for which reason the pellet fraction was used for analysis (1). On the other hand, MAP is an intracellular pathogen which in naturally contaminated milk is expected to be present inside the eukaryotic cells of the host and the portion of its free form is likely to be very low (19). The decision to use the pellet fraction of the sample for Actiphage-qPCR testing was supported by the optimisation study by Foddai and Grant (27) in which the vast majority of MAP was shown to sediment to the pellet after centrifugation at 2,500 g for 15 min. These conditions were thus used during milk sample preparation.

One problem we encountered when using Actiphage was clogging of the Rapid Tube column with cell debris (probably both eukaryotic and mycobacterial) and precipitated milk components present in samples, due to which the entire sample volume was not filtered through the column. To address this problem, we did not follow the BioSellal protocol according to which the prepared milk pellet is resuspended in Medium Plus with added bacteriophage D29 and immediately transferred to the upper part of the Rapid Tube, where the phage lysis of cells takes place. Instead, we decided to use the PBD Biotech manufacturer's instructions and, after resuspending samples in Medium Plus with bacteriophage D29, we incubated samples in 2-ml screw cap tubes. After a complete bacteriophage replication cycle and the full release of genomic DNA from all cells present in the sample, the supernatant was transferred into the top filter section of a Rapid Tube and centrifuged. As a result, we achieved the filtering of the whole sample through the column and avoided sample losses.

In general, estimations of MAP numbers present in milk specimens vary in different publications. While earlier studies focusing on MAP presence in milk documented low levels of MAP (2–8 CFU per 50 ml of milk) shed in milk and colostrum from both clinically and subclinically infected animals (28, 29), a more recent publication reported mean MAP shedding of 254 CFU/ml of colostrum in clinical animals and lower numbers (24 CFU/ml) in subclinical animals (30). In addition to the direct shedding of the pathogen in milk, environmental contamination by faecal material and poor udder hygiene may also play a role in the positivity of samples tested (31). Our results of MAP cell quantity estimations obtained by direct IS*900* qPCR (1.00 × 10^0^-2.11 × 10^1^ MAP per 50 ml-sample) corresponded to these studies. Taking into consideration the low MAP cell counts found in this kind of sample and the high sensitivity of the Actiphage determined, it can be assumed (due to its rapidity and the fact that the application of chemical sample decontamination is not necessary) that Actiphage may ensure more accurate viable MAP cell load determination in milk samples as compared to a culture and may enable a more reliable paratuberculosis status of animals to be established.

The effect of freezing and the use of preservative on the numbers of viable MAP in milk was evaluated previously by conventional PA and using artificial contamination of samples, with results showing the samples can be frozen at −70°C for up to 1 month without significant loss of MAP viability (27). However, limited information was available in the literature on the determination of MAP surviving by a phage-based assay in naturally contaminated frozen milk samples (8). Therefore, we subjected a total of 37 naturally contaminated samples that included direct IS*900* qPCR positive milk and milk filters frozen at −70°C for various lengths of time to testing by both Actiphage-qPCR and conventional PA (Table 3). From a practical perspective, the issue of MAP infectability by phage and the ability to determine viable MAP cells in samples after different freezing times by phage-based techniques is extremely important with regards to avoiding sample degradation, for example when the time until the testing of a sample is longer due to the transport of samples over longer distances. The positive Actiphage-qPCR result in a total of seven milk-based samples indicated that MAP cells retain their viability even during sample freezing and remain detectable by Actiphage-IS*900* qPCR even after long-term freezing (up to 7 years, i.e., more than required to transport frozen milk samples to the laboratory). On the other hand, given that the MAP load in the majority of specimens was low (1.00 × 10^0^-4.93 × 10^2^ cells per 50 ml milk and 1.09 × 10^0^-4.68 × 10^3^ cells per 1 g of milk filter, defined by direct IS*900* qPCR), this could be reflected in the negativity of some specimens (also if we consider that Actiphage-qPCR detects only the viable cell fraction within a sample). Using conventional PA, a total of 14 frozen milk-based samples produced plaques on an agar plate. However, most of them did not match the results of Actiphage-PCR, which can be explained by the uneven distribution of mycobacteria in the milk (11), the load of MAP cells in samples at the limit of isolation and detection methods, and the fact that not all plaques necessarily represent the presence of MAP. Considering these issues, it was previously generally recommended to test multiple samples (32).

The presence of environmental mycobacteria other than MAP was found in conventional PA-positive samples, which was revealed by a negative result of plaque PCR targeting IS*900* in a large number of plaques tested. In order to examine the functionality of the plaque PCR technique and to eliminate false negative results, the performance of three different plaque PCR procedures was analysed. All three plaque PCR procedures enabled to detected MAP DNA derived from a single plaque with a high sensitivity (Table 4) assuring us that plaque PCR applied after conventional PA allows good amplification of the IS*900* sequence and, therefore, MAP identification, consequently confirming that all these negatively tested plaques arose from other mycobacteria present in the samples.

This is the first demonstration of the application of the Actiphage-IS*900* qPCR method for viable MAP cell detection and quantification in milk samples. The combination of phage amplification with the IS*900* qPCR step proved to have great potential to selectively and with high sensitivity detect viable MAP in naturally contaminated milk samples from various animal species (cow, sheep, and goat). Not only is the commercial Actiphage-qPCR assay more sensitive than conventional PA and culture, it is much faster and easier to perform. Since the detection event of the assay is maintained in a single-tube format, automation of the procedure and high-throughput testing are possible. In addition, the test was also shown to be applicable to frozen milk samples, which is of practical relevance. However, due to the lack of correlation between the results of conventional PA and Actiphage-qPCR in frozen milk samples, further analysis of milk samples obtained from naturally infected animals is still needed to support the diagnostic utility of the Actiphage-qPCR method.

## Data Availability Statement

The original contributions presented in the study are included in the article/supplementary material, further inquiries can be directed to the corresponding author/s.

## Ethics Statement

Ethical review and approval was not required for the animal study because no tests on animals were conducted in this study, only milk samples, taken non-invasively, were examined.

## Author Contributions

MB designed and performed the experiments and wrote the manuscript. IS designed the study and revised the manuscript. All authors contributed to the article and approved the submitted version.

## Funding

This study was supported by grants from the Ministry of Agriculture of the Czech Republic (QK1910082 and RO0518).

## Conflict of Interest

The authors declare that the research was conducted in the absence of any commercial or financial relationships that could be construed as a potential conflict of interest.

## Publisher's Note

All claims expressed in this article are solely those of the authors and do not necessarily represent those of their affiliated organizations, or those of the publisher, the editors and the reviewers. Any product that may be evaluated in this article, or claim that may be made by its manufacturer, is not guaranteed or endorsed by the publisher.
